# Neuroadaptive modelling for generating images matching perceptual categories

**DOI:** 10.1038/s41598-020-71287-1

**Published:** 2020-09-07

**Authors:** Lauri Kangassalo, Michiel Spapé, Tuukka Ruotsalo

**Affiliations:** 1 Department of Computer Science, University of Helsinki, Helsinki, Finland; 2 Department of Psychology and Logopedics, University of Helsinki, Helsinki, Finland; 3 Helsinki Institute for Information Technology HIIT, Helsinki, Finland

**Keywords:** Computer science, Biomedical engineering

## Abstract

Brain–computer interfaces enable active communication and execution of a pre-defined set of commands, such as typing a letter or moving a cursor. However, they have thus far not been able to infer more complex intentions or adapt more complex output based on brain signals. Here, we present neuroadaptive generative modelling, which uses a participant’s brain signals as feedback to adapt a boundless generative model and generate new information matching the participant’s intentions. We report an experiment validating the paradigm in generating images of human faces. In the experiment, participants were asked to specifically focus on perceptual categories, such as old or young people, while being presented with computer-generated, photorealistic faces with varying visual features. Their EEG signals associated with the images were then used as a feedback signal to update a model of the user’s intentions, from which new images were generated using a generative adversarial network. A double-blind follow-up with the participant evaluating the output shows that neuroadaptive modelling can be utilised to produce images matching the perceptual category features. The approach demonstrates brain-based creative augmentation between computers and humans for producing new information matching the human operator’s perceptual categories.

## Introduction

Brain–computer interfaces aim to enable communication via a direct control pathway between the brain and an external device. For a long time, the attempts for such communication generally relied on explicit control of pre-specified commands, for example selecting letters in BCI spellers^1^ or cursor control^2^, rather than communicating with a more comprehensive model allowing the generation of new information matching the operators intentions. This is because inferring precise human intentions directly from the brain remains beyond the capabilities of the present imaging methods. At best, approaches attempting to ‘read the mind’ have been able to distinguish amongst clearly dissimilar categories, such as detecting whether a participant is thinking about animals or buildings^3^. Instead of trying to decipher the contents of the mind directly from the brain signals associated with thought processes, recent developments in brain–computer interfacing research have given rise to neuroadaptive technologies, in which the computer system models its user’s mental states using brain signals associated with stimuli^4,5^. While impressive, neuroadaptive BCIs have been successful only in narrowly constrained tasks, such as two-dimensional control of cursor movements^5^. Although learned from natural user responses, the approach is limited to controlling pre-specified parameters and does not allow adaptation to complex mental representations. To circumvent this need, recent neuroadaptive models have sought to utilize generative models^6,7^. However, their attempts were called into question due to confounds in the block-based experimental design, which overestimated the performance of the computational model^8^. It therefore remains unknown whether neurophysiological feedback can be harnessed to estimate user intentions toward mental categories by adapting generative models.

Here, we propose a novel modeling approach that combines a generative neural network with neuroadaptive brain interfacing. Instead of activating a limited set of pre-defined commands such as left or right cursor movements, the participant merely focusses on the goal of detecting images matching perceptual categories while passively watching images. The participant’s neural reactions are then used as feedback to parameterize a model of the user’s intention, despite the model’s architecture itself possessing no information on pre-defined stimulus categories. Then, the intention model can be used in generating previously non-existing images representing the perceptual categories of the operator.

To update the intention model of the user, we rely on detecting task relevance via classifying event-related potentials. Task relevance evokes a particularly strong pattern of neural activity that can be detected in EEG^9^. Indeed, BCI applications often rely on task relevance for controlling computers as it can be detected from the brain without requiring the user to perform any extraneous tasks such as performing motor imagery^10–14^.

We refer to the estimation of individual intentions by adapting a generative model to neural activity as *neuroadaptive generative modelling*. Our approach bears a resemblance to the neuroadaptive technology that is based on learning operator preferences from the variance in responses to stimuli which either match or violate the operator’s expectations^5^. However, instead of relying on pre-defined stimuli categories, we measure feedback directly from outputs of a generative model based on an adversarial neural network (GAN)^15^, which is able to generate highly realistic, yet artificial, digital information from a latent representation of an input space^16–18^. GANs learn to estimate the underlying distribution of input data, from which samples that may not be present in the original data can be generated. Instead of only learning to automatically label existing instances, these models can generate new instances from the learned distributions and produce previously unseen information that does not exist in the training data. GANs generate new information from a continuous, latent representation and thus there is a limitless number of possible samples that can be generated from it. By using brain activity to adjust a position in the latent space we are able to overcome the communicative restrictions of a system with pre-defined, fixed stimuli categories or operator states.

**Figure 1:**
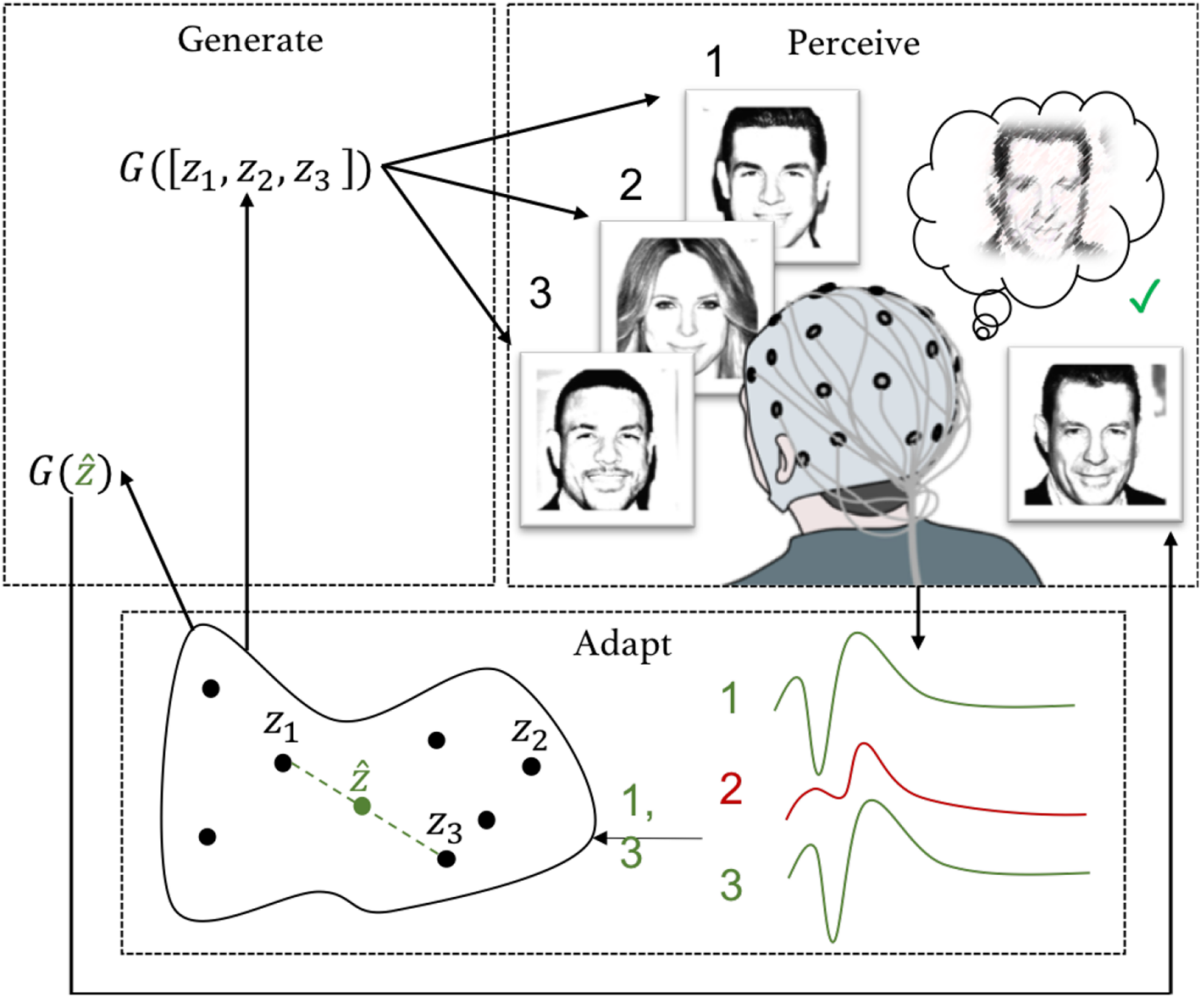
Overview of neuroadaptive generative modelling. Generate: the model *G* generates digital information based on latent variables $z_i$. Perceive: a human operator reacts naturally to the generated information represented by the computing system as $z_i$. Adapt: relevance of the information is inferred from the brain signals of the operator; the relevance guides the generative model, generating new digital information $G({\hat{z}}_n)$, which matches the operator’s perceptual categories. *n* is the number of acquired evoked brain signals, here $n=3$.

## Neuroadaptive generative modelling

Neuroadaptive generative modelling, as illustrated in Fig. 1, builds a model of its operator by testing computer-generated hypotheses on the operator. As the hypotheses are presented to the operator, the brain activity associated with the generated sampled output of the model (hypotheses) is used to update a model of the operator’s intentions. The underlying assumption is that after several iterations of observing user reactions to the generated samples, the intention model will converge to a state matching the operator’s mental target. As the model is generative, the approach allows to generate an output of the mental target that matches the operator’s intention.

Neuroadaptive generative modelling is based on three principles:

1. Generate: A generative model produces perceptually realistic and meaningful digital information to be used as sensory input.
2. Perceive: A human operator perceives and reacts naturally to the computer-generated sensory input.
3. Adapt: The task relevance is inferred from brain responses, which updates an estimate position in the latent generative model.

While using neuroadaptive generative modelling, the operator is performing a recognition task, such as “focus on the blond people you see”, on a set of generated hypotheses. The brain activity elicited upon encountering a target (a blond person) differs from the brain activity associated with non-targets. This difference is learned from the neural responses and utilized to update the model accordingly. Note that the model does not need to possess information of the task the user is performing. It will act only based on the difference of relevance and the corresponding variance represented by the generative model.

Formally, neuroadaptive generative modelling can be described as follows (see also Fig. 1). The generative model provides a mapping $G: Z \rightarrow X$, where $z \in Z$ is a point in a latent space *Z*, and $x \in X$ is digital information perceivable by humans (images of faces in the example). The goal of the system is to find a point ${\hat{z}}_n \in Z$, for which $G({\hat{z}}_n)={\hat{x}}_n$ matches the operator’s intention. To achieve this, a set of *n* images $X_n = \{x_1,\ldots ,x_n\}$ are generated from a set of latent representations $Z_n = \{z_1,\ldots ,z_n\}$. In Fig. 1 (perceive), this information is displayed to an operator, whose brain responses evoked by the presented information are measured. The presented $x_i$ will either match or violate the operator’s intention. In the example case, images 1 and 3 match, while image 2 violates the mental category.

As illustrated in Fig. 1 (adapt), the difference in brain activity evoked by matching and violating information is harnessed to update ${\hat{z}}_n$. More formally, the brain activity $S_n = \{s_1,\ldots ,s_n\}$ associated with the images is classified with a function $f: S \rightarrow Y$, where *Y* is the task-specific classification result for a brain signal (such as a binary value discriminating target/non-target stimuli). The classification results with their corresponding latent vectors are then used to compute a new latent representation ${\hat{z}}_n$. This is achieved with an intention model updating function $h: Z,Y \rightarrow Z$. Formally,

1 $$\begin{aligned} h({\varvec{Z_n}}, {\varvec{y_n}}) = {\frac{1}{\sum _{y_i \in {\varvec{y_n}}} y_i}}{\sum _{y_i \in {\varvec{y_n}},{\varvec{z_i}} \in {\varvec{Z_n}}}} y_i \cdot {\varvec{z_i}} = {\varvec{{\hat{z}}_n}}, \end{aligned}$$

where *n* is the iteration number, i.e. number of images shown to the participant, ${\varvec{Z_n}}$ is an $n \times m$ matrix consisting of *n* latent vectors of *m* dimensions ($m=512$ as per the model specification), and ${\varvec{y_n}}$ is an *n* dimensional vector, with each of the elements $y_i \in \{0,1\}$ representing the classification result (non-target/target) for the *i*th stimulus. The algorithm is a special case of the Rocchio algorithm^19^. The final output image is generated from ${\hat{z}}_N$, where *N* is the total number of images displayed during a feature recognition task.

**Figure 2:**
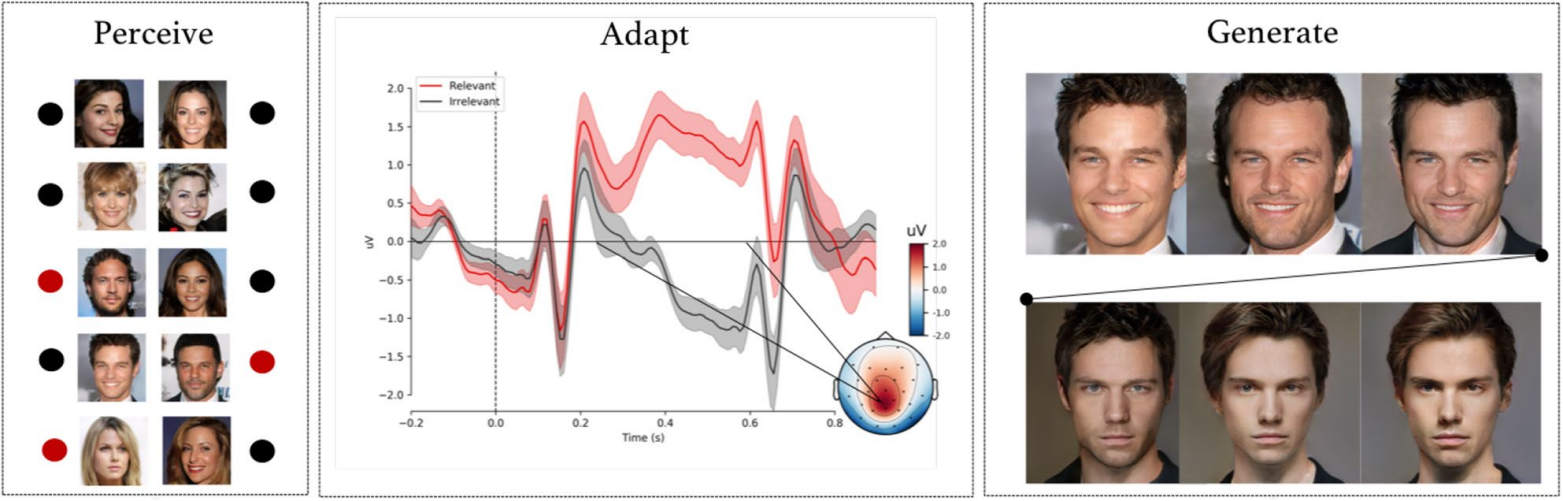
Example data in the human face generation experiment. Perceive: an extract of images shown to a participant during the ‘no smile’ task; here, task-relevant images are marked with a filled red circle; adapt: an average ERP from the Pz-electrode (top) with 95% confidence intervals, and a topographic plot of the difference of relevant and irrelevant evoked response for the 250–600 ms post-stimuli interval (bottom); both averaged over all tasks and participants; the difference between relevant and irrelevant classes was utilized by a classifier to adapt the generative model; generate: resulting generated mental target visualizations $G({\hat{z}}_n)$ for the ‘no smile’ task for a participant after displaying $n=$ 5, 10, 15, 50, 100, 200, and 242 (all) images; the final image matches the image in Fig. 3 in the second column of the ‘no smile’ row.

The resulting ${\hat{z}}_n$ can then be used as an input to the latent model *G* to generate a new image based on the brain signals of the participants, $G({\hat{z}}_n) = {\hat{x}}_n$. The images in Fig. 2 (right) are the generated images ${\hat{x}}_n$ for various *n* and display the model’s convergence towards the mental target.

To summarize the formalization of neuroadaptive generative modelling with regards the three parts:

1. Generate: A latent model $G: Z \rightarrow X$, which provides a mapping from a latent space *Z* to an image space *X*.
2. Perceive: A participant who views images $X_n$ while their brain signals $S_n$ are measured.
3. Adapt: A brain signal classifier $f: S \rightarrow Y$, where *Y* is the predicted relevance of the stimulus; and an intention model updating function $h: Z,Y \rightarrow Z$ mapping the predictions made by the classifier to the latent space *Z*.

To update ${\hat{z}}_n$, we harnessed the known effect of relevance^20^ on brain activity as measured using event-related potentials (ERPs) evoked in response to the displayed images. The P300 is a late parietal positivity in the EEG that is amplified by infrequent, attended, novel, and task-relevant stimuli, regardless of their modality^21,22^. Dominating psychophysiological theories suggest the P300 signifies enhanced cognitive processing of presented stimuli, whether as a function of working memory updating^23^, or for attention and further memory processing^20^. Here, we expected target feature relevance to amplify the P300. Our approach relies on attention allocation of the participants on visual features defining a category within stimuli. Notably, however, we do not require participants to perform any artificial background task, such as motor imagery or explicit counting of relevant stimuli, but simply pay attention to the target category.

## Experiment

To test the validity of the neuroadaptive generative modelling approach, we present a practical implementation and report a two-phase experiment to empirically evaluate the neuroadaptive model’s performance. In the first phase (data acquisition), participant’s EEG responses toward the generated images were recorded, models representing each participant’s mental intentions were constructed and then used to generate images matching the target categories without the participants having explicitly communicated any such information. In the second phase (validation), run separately between 1 and 3 months after the first phase, the participants were called back to the laboratory and generated images were presented to them along with control images produced by a simulation in which random feedback and negative (the images classified as irrelevant) feedback were given to the same model. The participants were then asked to select and judge the generated images and the controls according to how well they matched the target perceptual categories.

In detail, the first phase of the experiment (data acquisition) consisted of four parts. First, a set of stimuli images were generated using a pre-trained GAN *G* (see^24^ and section “Generative latent model” in “Materials and methods” for model specification). A fair, representative sample was obtained to ensure coverage of the entire GAN space and to avoid over-representation of any specifc task category that could in turn lead to local maxima convergence (see section “Stimuli” in “Materials and methods” for details).

Second, the images were presented to 31 participants in a rapid serial visual presentation sequence while their EEG was recorded. The participants completed eight different facial cateogry recognition tasks in which they were asked to focus on faces that matched the intended task category: were male, were female, were young, were old, were smiling, were not-smiling, had blond hair or had dark hair.

Third, the participants’ evoked brain responses were split to training and testing data separately for each participant and task with 80/20 split, respectively. The data split followed the stimuli presentation structure to mimick online execution so that feedback was given in the order in which the corresponding images were seen by the participants. This allowed us to simulate the generation process as it would happen within an on-line experiment, but with varying feedback.

Fourth, the classifier was used to classify the evoked brain responses in the testing set into relevant and irrelevant images. As each of the stimuli images were sampled from the GAN space, they were associated with a correspoding latent vector *z*. For the positive model, the vectors corresponding to relevant images were used to adapt the intention model ${\hat{z}}_n$ and to generate a visualisation of their mental target (${\hat{x}}_n$). Figure 2 (perceive) shows examples of images shown during the ‘no smile’ task.

As any generated face could match the intended task category by chance and the lower bound of the performance is unknown, three generative feedback models were compared: positive (maximally positive estimate in which only the latent vectors for images classified as relevant were used as an input), negative (maximally negative estimate in which only the latent vectors of images classified as irrelevant were used as an input) and random (the same amount of feedback was given as in the positive model, but with their labels shuffled in accordance with the permutation test protocol^25^ (further specified in “Materials and methods”6). Training data was then used to calibrate a classifier to detect brain responses for relevant and irrelevant images.

In the second phase of the experiment (validation), a validation study was conducted to measure the performance of the positive model (relevant feedback) against negative (irrelevant feedback) and random (random feedback). Two tasks were used in the validation study: a selection task and a rating task. In the selection task, the participants were first presented with the generated image from the positive model, with the generated image from the negative model, and with twenty images resulting from the random model. These were displayed simultaneously, randomly laid out within an image grid. The participants were asked to select all images that matched the task (see Figure S3 in the supplementary information). Then the same images from the three models were presented sequentially one at a time in a randomized order and the participants were asked to rate each image on a Likert scale according to how well they matched the task (see Figure S4 in the supplementary information). This allowed to directly compare how often the positive model output was selected and rated compared to negative and random model outputs. The validation study followed a double-blind procedure. That is, neither the participants were aware of which of the faces were the ones generated from positive, negative, or random feedback, nor was the laboratory assistant conducting the study.

The performance of the neuroadaptive model’s image generation was then quantified using two measures; one for each task. The first measure was the likelihood of participants selecting the generated images and the second measure was the mean rating.

In the following section, we report the results. We first verify known effects of stimuli matching relevant features on the averaged ERP. We then show that the classifiers find meaningful structure from the data. Finally, we show that the generated images were perceived as matching the target features using the data from the validation study.

## Results

Event-related potential analysis was conducted to verify that target relevant features modulated evoked brain activity to the extent that this could be used for updating ${\hat{z}}_n$. The analysis revealed that task-relevant stimuli images were associated with parietal positivity from ca. 250 ms following stimulus onset. As shown in Fig. 2 (adapt), the positivity was maximal at 464 ms (mean difference = 2.36 $\upmu$V, SE = 0.20 $\upmu$V), continuing until well after presentation of the subsequent stimulus. Offline analysis of task-related effects suggested that the latency of the potential depended on the task, though generally occurring between 250 and 450 ms. As these findings correspond with the literature on the P300 with regards to latency, topography, and task-dependence, we identified the grand average effect with the P300.

**Figure 3:**
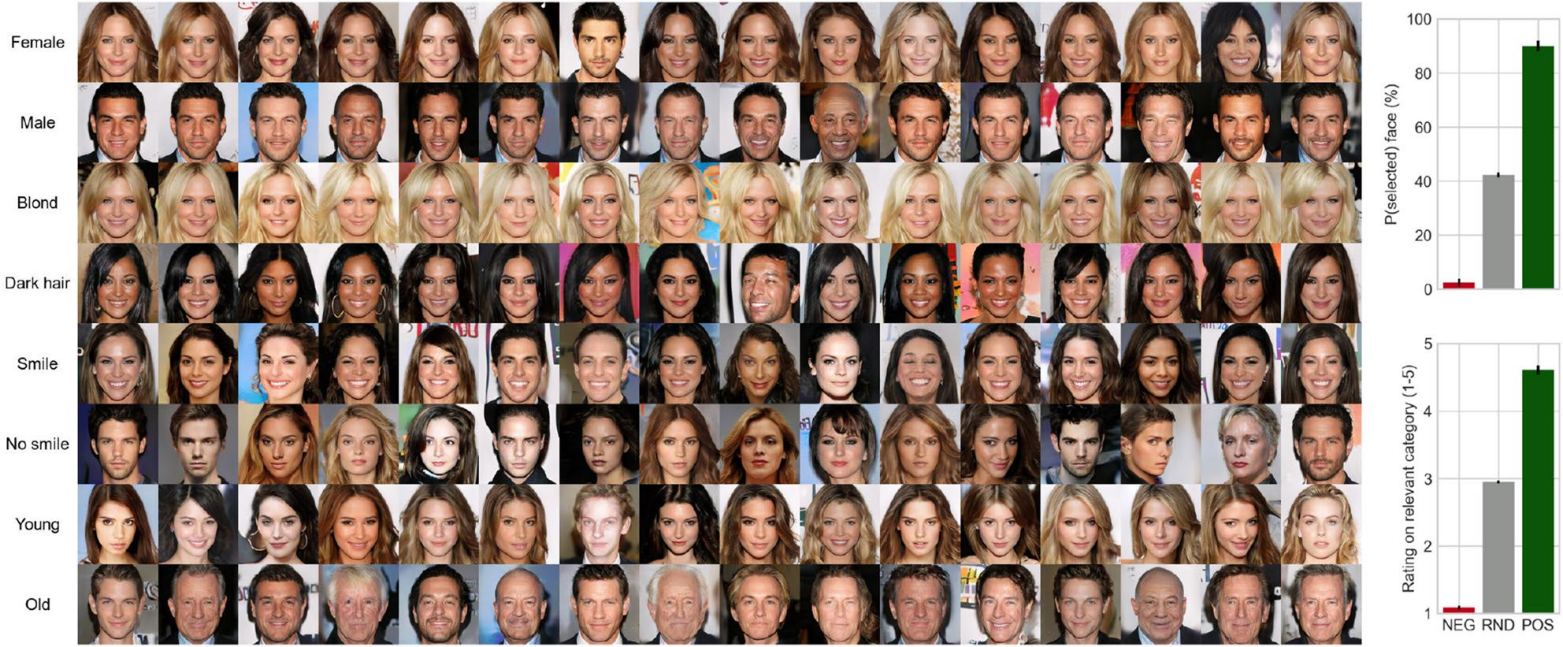
Left: generated images for 16 participants and all tasks; right top: percentages and standard errors of resulting images chosen to match task criteria; right bottom: mean ratings and standard errors for a resulting image to have a task-relevant feature; resulting image labels: *NEG* images generated from negative predictions, *RND* images generated using the same process, but with random feedback, *POS* images generated from positive feedback. The number of positively classified images and the corresponding latent latent vectors used as feedback varied between 5 and 60, depending on participant and task.

To utilize the pattern presented within the P300 to adapt the generative model, linear classifiers were trained to predict the task-relevant samples (e.g. images of blond-haired persons in the blond task) from evoked brain activity. The classifiers performed with relatively high AUC scores for all participants (mean AUC = 0.789, p < 0.05, see Supplementary Figure S1 for per-participant AUC scores). The latent vectors corresponding to the positive predictions were used to form a new latent vector ${\hat{z}}_n$, from which an improved image matching the participant’s intention $G({\hat{z}}_n)$ was generated for each participant and task. Figure 2 (generate) shows a sequence of generated images for the ‘no smile’ task during the course of the task for one randomly selected participant. As more stimuli images are shown, the intention model starts to converge towards a non-smiling person (see Supplementary Movie S1 for an animation of the convergence). Figure 3 displays the final generated images of all of the tasks for randomly selected 16 participants (see Supplementary Figure S2 for results of all participants). The facial features in the images correspond to the associated task for nearly all tasks and participants.

To evaluate the generated images, we requested participants to perform two validation tasks. In the first validation task, participants were shown images from the negative model, positive model, and 20 randomly generated control images for each task, and were asked to select every image that matched the perceptual category, thus depending on the task selecting faces that were blond, dark-haired, male, female, young, old, smiling, or not smiling (see Supplementary Figure S3 for a screenshot of the system used for the validation test). As shown in Fig. 3 (top right), this double-blind procedure validated that the generated images matched the intention specified in the task. The image from the positive model (only positive predictions as feedback) was chosen on average 90%, the negative 2.5%, and the random 42.3% of trials. Binomial tests on the positive model showed this generalised across tasks, with the lowest performance for the positive model of not smiling (76.67%, p = 0.003, Bonferroni corrected p = 0.021).

In the second validation test, participants were requested to make explicit evaluations of the generated images using Likert-type rating scales on the relevant categories (e.g. how old do you find the face in the picture on a scale from 1 to 5; see Supplementary Figure S4 for a screenshot of the validation test). The images generated based on the positive model were rated on average 4.61 (± 0.37), negatives 1.09 (± 0.14) and random 2.95 (± 0.13). To statistically test the results, a repeated measures ANOVA with task (blond, dark-haired, male, female, young, old, smiling, not-smiling) and generative model output (negative, random, positive) on the average ratings of the generated images was conducted. This showed significant effects of the generative model output, F (7, 203) = 1216.37, MSE = 0.61, p < 0.0001, eta-sq = 0.83. As shown in Fig. 3 (bottom right), the effect was very strong, with negative (M = 1.09, SE = 0.04) and positive (M = 4.61, SE = 0.04) feedback models performing near floor and ceiling respectively. There was also a significant effect of task, F (7, 203) = 8.84, MSE = 0.33, p < 0.0001, eta-sq = 0.01, showing generally slightly higher ratings on the dark-haired evaluations, and lowest on the no-smile ones. Finally, the interaction was also significant, F (14, 406) = 8.35, MSE = 0.34, p < 0.0001, eta-sq = 0.02, with differences between positive and random models being smaller for the young and no-smiling tasks, and larger for blond and male tasks.

## Discussion

We introduced neuroadaptive generative modelling as an approach to generate images matching perceptual categories by adapting a neural network model to brain signals. To the best of our knowledge, this is the first study to use neural activity to adapt a generative computer model and produce new information matching a human operator’s intention. Previous attempts in neuroadaptive brain–computer interfaces^5,26^ already take advantage of designing goal-oriented control of a computer system by loosely relying on natural reactions to perception. While impressive, neuroadaptive BCIs have been successful only in narrowly constrained tasks, such as for two-dimensional control of cursor movements^5^. To our knowledge, such BCIs have not been utilized to generate sophisticated digital information, such as images matching human expectations.

An advantage of neuroadaptive generative modelling is that it enables reactions evoked by natural stimuli to be mapped to complex features learned by a generative model without repeating the same stimuli or requiring the operator to perform artificial imagery tasks. Instead, the stimuli are represented by latent vectors and machine-learning can be used to update a vector representing the operator’s intention using the latent feature space. The approach allows to probe the operator’s responses to very high-dimensional latent vectors in a way that the resulting changes in the features can easily be parsed by the operator. Thus, the approach is not limited to a pre-defined set of commands, but updating a model of the operators intention over a generative model allows focusing on any visual features producable from the generative model.

Our experiment provided strong evidence that neuroadaptive modelling is highly effective in generating previously non-existing information matching the human operator’s intended perceptual categories. The resulting images achieved nearly perfect agreement between the neuroadaptive generative modelling output and post-experiment human judgement and were shown to significantly outperform random and a generative processes with negative feedback by a large margin. While, presently, the studied visual features were purposefully straightforward (such as gender, hair color, age, and smile) and in a relatively restricted domain (human faces), the results show that the neuroadaptive generative modelling paradigm can be used to gather information on highly complex, subjective concepts, such as specific facial features.

When devising a neuroadaptive generative model, one should take in to account the bias introduced by the selected generative model. By design, the generative model produces samples whose feature distribution corresponds to that in its training set. For instance, the current model was trained with celebrity data, relatively overrepresenting smiling faces. This bias in the model may explain the variance in performance across tasks; the ‘no smile’ task had a lower accuracy than the ‘smile’ task. Alternatively, the differences in performance can be explained by the subjective perception of facial features, such as what constitutes a smile.

Due to the selection of tasks, some of the features in the mental target visualisations could be explained by lower-level features found in the images, such as the difference in luminance between the blond/dark-haired tasks. This underlines an important feature of the neuroadaptive generative model: although on average, the results show the largest differences in the P300 potential, the machine learning uses all available signal features that are useful for the task. Thus, for example, very early potentials such as the P100 and N1 may detect relative luminance levels while the face-sensitive N170 potential can reliably detect facial features^27,28^. The N170 is also known to be amplified with expressions such as smiles^29^, and occurs even in the absence of conscious perception^30^. For such features, the neuroadaptive model could theoretically predict mental categorisation without placing any cognitive demands on the operator. However, detecting a more complex visual feature, such as perceived gender or age, may require the full processing as indexed by the P300.

Our implementation of the neuroadaptive generative model is based on the BCI research tradition of classifying ERPs in response to stimuli. However, unlike BCIs, our approach focusses on modelling human perceptual categorisation rather than communicating commands to a computer. Thus, unlike systems for brain-control or neuroadaptive brain–computer interfaces, our approach does not rely on repetitive single-trial target classification to communicate letters or movements. Instead, the neuroadaptive generative model learns relevance from ERPs and iteratively adapts an intent model over a GAN space to infer images matching perceptual categories. The participant’s intention is thereby modelled without a need of a priori labelling of the data or stimuli. The paradigm is therefore not restricted to either EEG or GANs, but could learn from any implicit or explicit feedback and use any model providing a sufficiently complex representational space.

While at present computational requirements of image generation with GANs prevents us from creating a closed-loop BCI design and allow only off-line experimentation, the current study provides foundational elements to guide a future implementation. Such a system would use a similar design as the one presented to obtain a selection of generated images using on-line model updating via relevance feedback^19^, Bayesian optimization^31^, or on-line reinforcement learning^32^. In terms of implementation, the design could complement the task-relevance related signals with error detection^33–35^, providing feedback towards future avoidance of undesired behaviour of the generative model. Indeed, a BCI based on neuroadaptive generative modelling could harness a combination of stimulus selective activity, relevance related positivity, and error related negativity to respectively select and test competing hypotheses generated by the generative model in an exploration/exploitation loop.

The implications of our work are therefore broader than our experimental validation may suggest. The general effectiveness of human–machine interaction today is largely based on explicit command and control in which humans are required to translate high-level concepts into explicit machine-understandable commands. While in the case of brain–computer interfaces these commands are transmitted implicitly, the mental imagery is explicit and often artificial. For example, the operator may be required to perform motor imagery by imagining moving an arm^36^. Such interfaces may perform well if they rely on tasks that allow direct mapping of the mental onto the physical task, but fall short with tasks requiring higher-level cognition. At best, passive brain–computer interfacing and neuroadaptive methodologies have shown potential to learn these patterns implicitly for simple tasks, such as cursor control^5,26^. In contrast, our approach demonstates that coupling brain–computer interfaces with generative models allows human–machine symbiosis that is capable of learning a representation of human intention and goes well beyond transmission of simple commands.

We also believe that the neuroadaptive generative modelling approach presents a new paradigm that may strongly impact experimental psychology and cognitive neuroscience. The neuroadaptive model constitutes a novel methodology that may inform on ongoing debates on the nature of mental representation^37^, and whether representations are based on stereotypes, family resemblances, symbolic descriptions, or depictions. That is, the model is not necessarily limited to easily identifiable, objective features, but can utilise brain potentials evoked by more abstract, culturally understood features. For example, the literature on brain potentials that are sensitive to subjective features such as familiarity^38^, attractiveness^39^ or social dimensions^40^ may inspire the design of neuroadaptive models that can generate empirically verifiable visualisations of subjective features. In other words, the generative functionality of the neuroadaptive modelling approach not only promotes augmentation of creative interaction between computers and humans, but also opens new avenues for neurophysiological research into how perceptual information is represented in the human brain.

## Materials and methods

### Neurophysiological experiment

This section describes the neurophysiological experiment undertaken to acquire the data used for the validation of the neuroadaptive generative modelling approach.

#### Participants

Thirty-one volunteers were recruited for the study using convenience sampling from the undergraduate and postgraduate student population of the University of Helsinki. Of these, one left before completing all tasks and was removed from analysis. The rest comprised 17 males and 13 females, with an average age of 28.23 (SD = 7.14, range 18–45). The study was approved by the University of Helsinki Ethical Review Board in the Humanities and Social and Behavioural Sciences. Participants received full instruction as to the nature and purpose of the study, and were fully informed as to their rights as human participants in agreement with the Declaration of Helsinki, including the right to withdraw at any time without fear of negative consequences. In return for their participation in the data acquisition part of the study, they received one cinema voucher, and another two after returning for the validation part.

#### Stimuli

The stimuli images were generated with the following process. 70,000 latent vectors were sampled from a 512-dimensional multivariate normal distribution, and their corresponding images were generated with the latent model. The sampling procedure ensured that the images represented the entire GAN space, but did not overrepresent any particular subspace. Then, the images were filtered to remove artefacts and sorted to eight categories (female, male, blond, dark hair, smile, no smile, young, old) by a human assessor, resulting in a set of 1961 stimulus images. To standardise the generated 1024 $\times$ 1024 pixels sized stimuli thus obtained to minimalize contribution of physical characteristics unrelated to the face (e.g. background), we applied a 746 $\times$ 980 silhouette cutout with the surrounding area made uniform grey (RGB 125, 125, 125). The images were then downsampled to a resolution of 512 $\times$ 512 pixels for data acquisition timing purposes, and presented at a distance of approximately 60 cm on a 24” LCD monitor running a resolution of 1920 $\times$ 1080 at 60 Hz. Image randomisation, trigger synchronisation, and response collection was handled via E-Prime 3 (Sharpsburg, PI).

#### Data acquisition procedure

The feature recognition task started after the participants signed informed consent. This part comprised 8 blocks across which the task was randomised between categories of relevant stimuli (female, male, blond, dark hair, smile, no smile, young, old). Each block comprised 4 rapid serial visual presentation (RSVP) trials during which 20 relevant and 50 irrelevant stimuli were presented. For each task, irrelevant stimuli were always sampled from the set comprising the complementary category to the relevant task (e.g. old if young is relevant). At the beginning of the RSVP trial, participants were reminded to passively watch the images but concentrate specifically on those they noticed belonging to the relevant category. To demonstrate the task, they were also shown 4 unique stimuli, 2 of which were sampled from the relevant, 2 from the irrelevant sets, and asked to click on a relevant image. Following a 1,000 ms blank screen, the RSVP trial commenced, in which images were presented at a constant pace of 2 Hz (500 ms) without inter-stimulus interval. They were sampled randomly in groups of five with the following restrictions: no (a-priori) relevant stimulus followed another relevant stimulus, and in any sequence of five stimuli, at least one was relevant. A blank 500 ms inter-trial interval, followed by a self-terminated warning for the next, ended the trial. The experiment, including setup, took ca. 1 h to complete.

#### Validation procedure

All participants returned between 1 and 3 months after the data acquisition part of the study. Following signing of informed consent, participants completed 4 blocks across which the relevant and irrelevant categories were combined to form four pairs: smile vs no smile, blond vs dark-haired, young vs old, and male vs female. Within each block, two tasks were presented in sequential order. In the first task, 24 images were presented simultaneously across two rows of 12, and participants were requested to click on every image fulfilling one of the categories. Of the 24, 2 were generated from the positive model (relevant feedback), 2 were generated from the negative model (irrelevant feedback), and 20 were generated from the random model. Subsequently, they were requested to perform the same task, but for the complementary category. We analysed the percentage of times an image from the positive, random, and negative models were chosen or not chosen. In the second task, the 48 earlier presented images of the two categories were displayed in random order along with 1–5 rating scale. Following completion of all tasks across all four blocks, the participants were shown their generated images and a debriefing concluded the experiment.

#### EEG data acquisition and preprocessing

EEG was recorded from 32 Ag/AgCl passive electrodes with initial ground/reference at AFz, positioned on equidistant sites from the 10/20 system using an elastic cap (EasyCap). A BrainProducts QuickAmp USB was used to digitise the electric potential a sample rate of 1,000 Hz, with hardware applying a 0.01 Hz low-cut filter and an average re-referencing. To remove slow signal fluctuations and high-frequency noise from the EEG recordings, the measured EEG data were band-pass filtered for the frequency range 0.2–35 Hz with a Fir1 filter. After filtering, the data were split to baseline corrected epochs ranging from − 200 to 900 ms time-locked to stimulus onset. A simple threshold-based heuristic was used to remove transient artefacts from the data, such as those caused by eye blinks. This led to the removal of approximately 11% of each participants’ epochs with the highest absolute maximum voltage. Finally, the data was decimated with a factor of four to speed up classifier training procedures. The final dataset consisted of on average 3,251 epochs per participant. Supplementary Table S1 provides per-participant recorded/dropped epoch counts and voltage threshold values used for removing contaminated epochs.

### Neuroadaptive generative modelling implementation

This section follows the formal definition of the neuroadaptive generative modelling approach. It defines the latent model *G*, brain signal classification function *f*, and the intent model updating function *h*. Additionally, the classification performance tests are described.

#### Generative latent model

A pre-trained Generative Adversarial Network (GAN) was used to generate the face images^24^(source code and pre-trained models are available at: https://github.com/tkarras/progressive_growing_of_gans). Essentially, GANs consist of a generator (*G*) and a discriminator (*D*)^15^. During the training of a GAN, *G* and *D* are trained simultaneously, so that the objective of *D* is to determine whether its input is from the original training set or not. Conversely, *G* tries to “fool” *D* by generating output resembling the original training set more closely. Feeding *G*’s output to *D* as input results in a game between *D* and *G*, which can be leveraged to train the generator to produce high-quality output from an internal representation (latent space). The GAN used in this study was pre-trained with the CelebA-HQ dataset, which consists of 30,000 $1{,}024 \times 1{,}024$ images of celebrity faces^24^. The CelebA-HQ dataset is a resolution-enhanced version of the CelebA-dataset^41^. The generator part *G* of the aforementioned GAN provided the mapping $G: Z \rightarrow X$, where $z \in Z$ is a 512-dimensional latent vector and $x \in X$ is a $1{,}024 \times 1{,}024$ image.

#### Brain signal classification

The classification function $f: S \rightarrow Y$ was implemented with Regularized Linear Discriminant Analysis (LDA) classifiers^42^ trained for each of the participants. The regularization parameters for the classifiers were chosen with the Ledoit–Wolf lemma^43^. The classifiers were trained with vectorized representations of the ERPs ($S_n$) along with a binary label indicating class membership (relevant/irrelevant for task). The vectorized representation of the ERPs consisted of spatio-temporal features, namely all available 32 channels and 7 averaged equidistant time-windows in the 50–800 ms post-stimuli interval. A classifier was trained for each participant and task separately. The task-specific classifier was trained with data collected during all of the tasks performed by the participant, excluding the reverse task. For instance, a classifier predicting the labels for the blond task was trained with data from the tasks male, female, young, old, smile, and no smile. The reverse task was excluded from the training set to ensure that the training and test sets do not contain brain responses for the same stimuli images. To reduce the number of false positives, only predictions with a confidence score exceeding 0.7 for the relevant class were considered positive. The positive predictions received a value of $f(s) = 1$, while the negative predictions received a value of $f(s) = 0$. Thus, $Y = \{0,1\}$.

#### Classifier evaluation

The classifier performance was measured with an Area Under the ROC Curve (AUC), and evaluated by permutation-based p values acquired by comparing the AUC scores to those of classifiers trained with randomly permutated class labels^25^. $k = 100$ permutations were run per participant, leading to a minimum possible p value of 0.01^44^. The AUC scores of the classifiers can be seen in Supplementary Figure S1.

## Supplementary information

Supplementary material 1

Supplementary material 2

## Data Availability

The datasets generated during and/or analysed during the current study are available from the corresponding author on reasonable request.
