# Supplementary material for: Neuroadaptive modelling for generating images matching perceptual categories
Source: Sci Rep. 2020 Sep 7;10:14719. doi: 10.1038/s41598-020-71287-1 (PMC7477223; doi:10.1038/s41598-020-71287-1)
Supplement: Supplementary file 2 — Supplementary material 2 [file 41598_2020_71287_MOESM2_ESM.pdf]

Supplementary Information for

Neuroadaptive modelling for generating images matching perceptual categories

Lauri Kangassalo, Michiel Spapé, and Tuukka Ruotsalo

Tuukka Ruotsalo
E-mail: tuukka.ruotsalo@helsinki.fi

This PDF file includes:

Figs. S1 to S4
Table S1
Caption for Movie S1

Other supplementary materials for this manuscript include the following:

Movie S1

Participant	Recorded epochs	Cleaned epochs	Threshold (μV)
01	3678	3059	80.00
02	3680	3396	69.66
03	3679	3111	41.20
04	3680	3302	34.75
05	3681	3309	27.36
06	3679	3354	56.02
07	3678	3444	80.00
08	3679	2931	80.00
09	3680	3406	74.23
10	3678	3277	36.25
11	3679	3308	51.84
12	3679	3369	30.18
13	3679	3376	41.61
14	3679	3393	37.19
15	3679	2767	80.00
16	3679	3292	40.43
17	3677	3203	80.00
18	3680	3283	27.52
19	3680	3329	51.93
20	3679	2874	36.17
21	3680	3414	34.01
22	3680	3342	80.00
23	3679	3343	30.80
24	3680	3260	80.00
25	3679	3310	56.05
26	3678	3426	57.98
27	3678	3342	36.45
28	3679	3323	77.94
29	3680	3179	40.58
30	3680	3116	80.00
31	3336	2969	49.37

Table S1. Details of EEG preprocessing. Epochs with a maximum absolute voltage exceeding the per-participant voltage threshold were removed. The threshold was limited to a maximum of 80 μV.

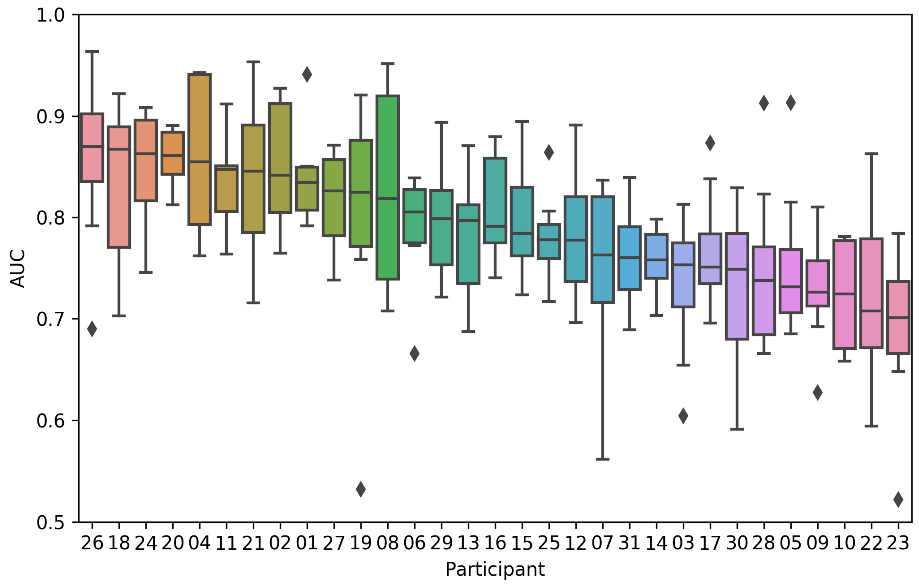

Fig. S1. AUC scores of all classifiers.

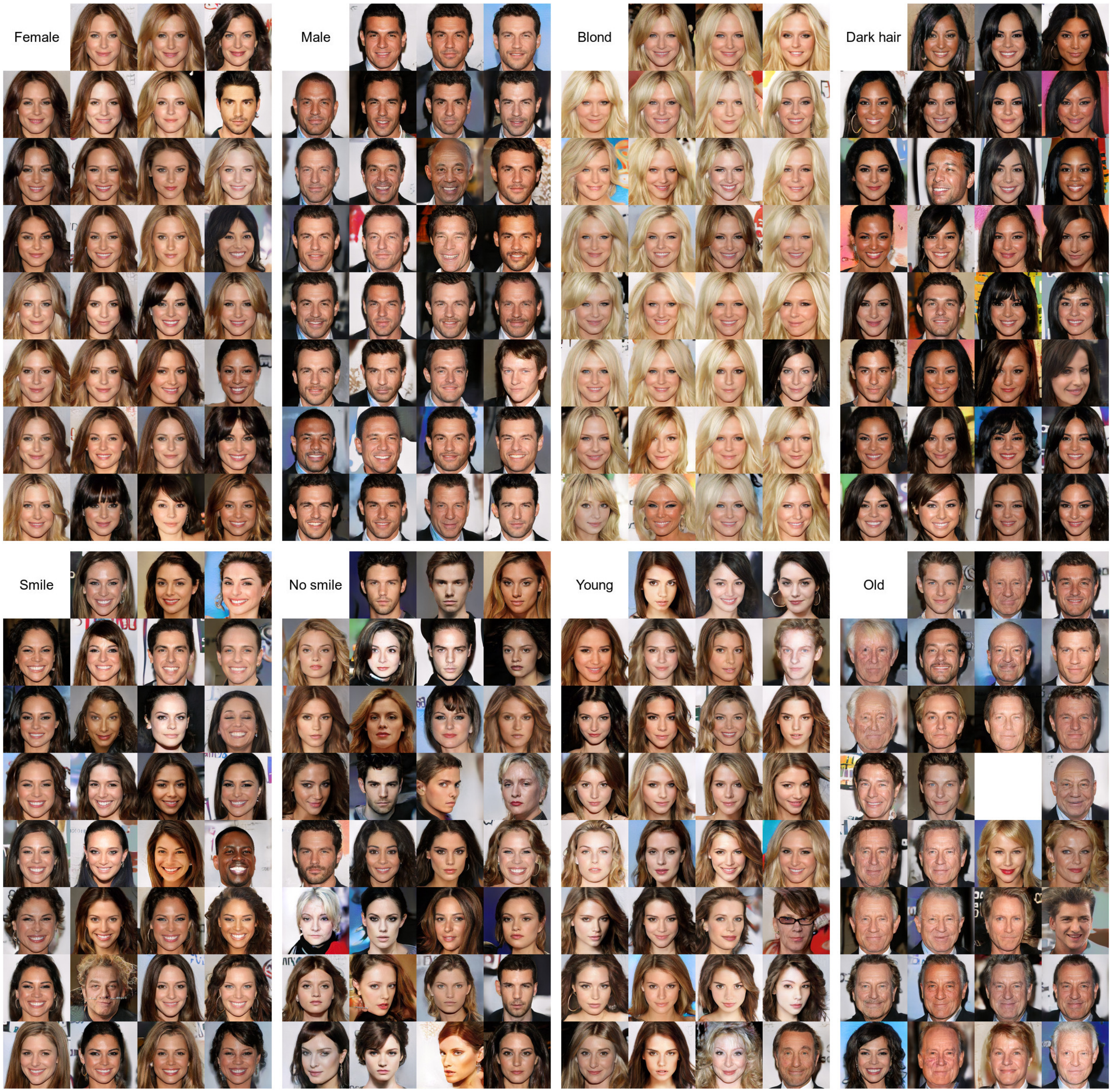

Fig. S2. Generated mental target visualisations for all participants and tasks. The images in each task are ordered by participant identifier from left to right, top to bottom. For instance, the image in the first column of the second row in all of the tasks was generated for participant 04. Participant 14's 'old' task was omitted due to technical problems.

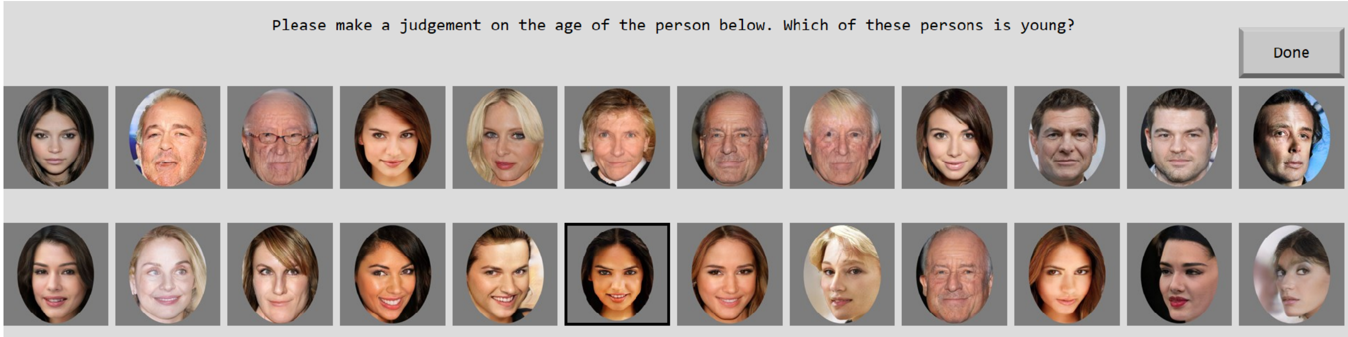

Fig. S3. A screenshot of the free selection validation task for the feature recognition task 'young'.

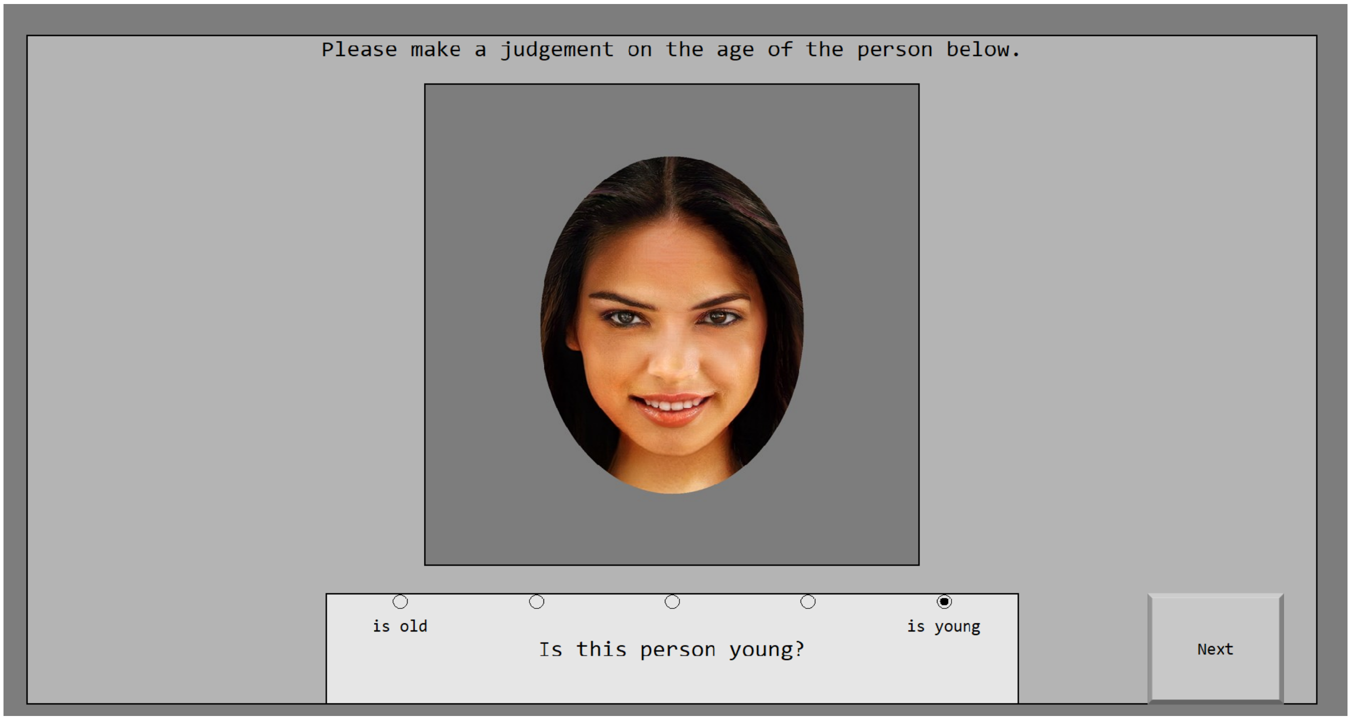

Fig. S4. A screenshot of the rating validation task for the feature recognition task 'young'.

Movie S1. Convergence of the mental target visualisation for a random participant completing the 'no smile' task. The video has been interpolated by adding 15 frames between each update of $G(\hat{z}_n)$ (i.e. each positive prediction of the classifier).
